# Supplementary material for: Galectin-9 Triggers Neutrophil-Mediated Anticancer Immunity
Source: Biomedicines. 2021 Dec 29;10(1):66. doi: 10.3390/biomedicines10010066 (PMC8772786; doi:10.3390/biomedicines10010066)
Supplement: Supplementary file 1 [file biomedicines-10-00066-s001.zip › biomedicines-1512251-supplementary.pdf]

Galectin-9 triggers neutrophil-mediated anticancer immunity

Natasha Ustyanovska Avtenyuk [1,†], Ghizlane Choukrani [1,†], Emanuele Ammatuna [1], Toshiro Niki [2], Ewa Cendrowicz [1], Harm Jan Lourens [1], Gerwin Huls [1], Valerie R. Wiersma [1,*,†] and Edwin Bremer [1,*,†]

[1] Department of Hematology, University Medical Center Groningen (UMCG), University of Groningen, 9713 GZ Groningen, The Netherlands.n.ustyanovska.avtenyuk@umcg.nl (N.U.A.); g.choukrani@umcg.nl (G.C.); e.ammatuna@umcg.nl (E.A.); niki.toshiro@kagawa-u.ac.jp (T.N); e.krol@umcg.nl (E.C.); h.j.lourens@umcg.nl) (H.J.L.); g.huls@umcg.nl (G.H.)

[2] Department of Immunology, Kagawa University, Takamatsu, Kagawa 760-0016, Japan

* Correspondence: e.bremer@umcg.nl (E.B); v.wiersma@umcg.nl (V.R.W.).

† These authors contributed equally.

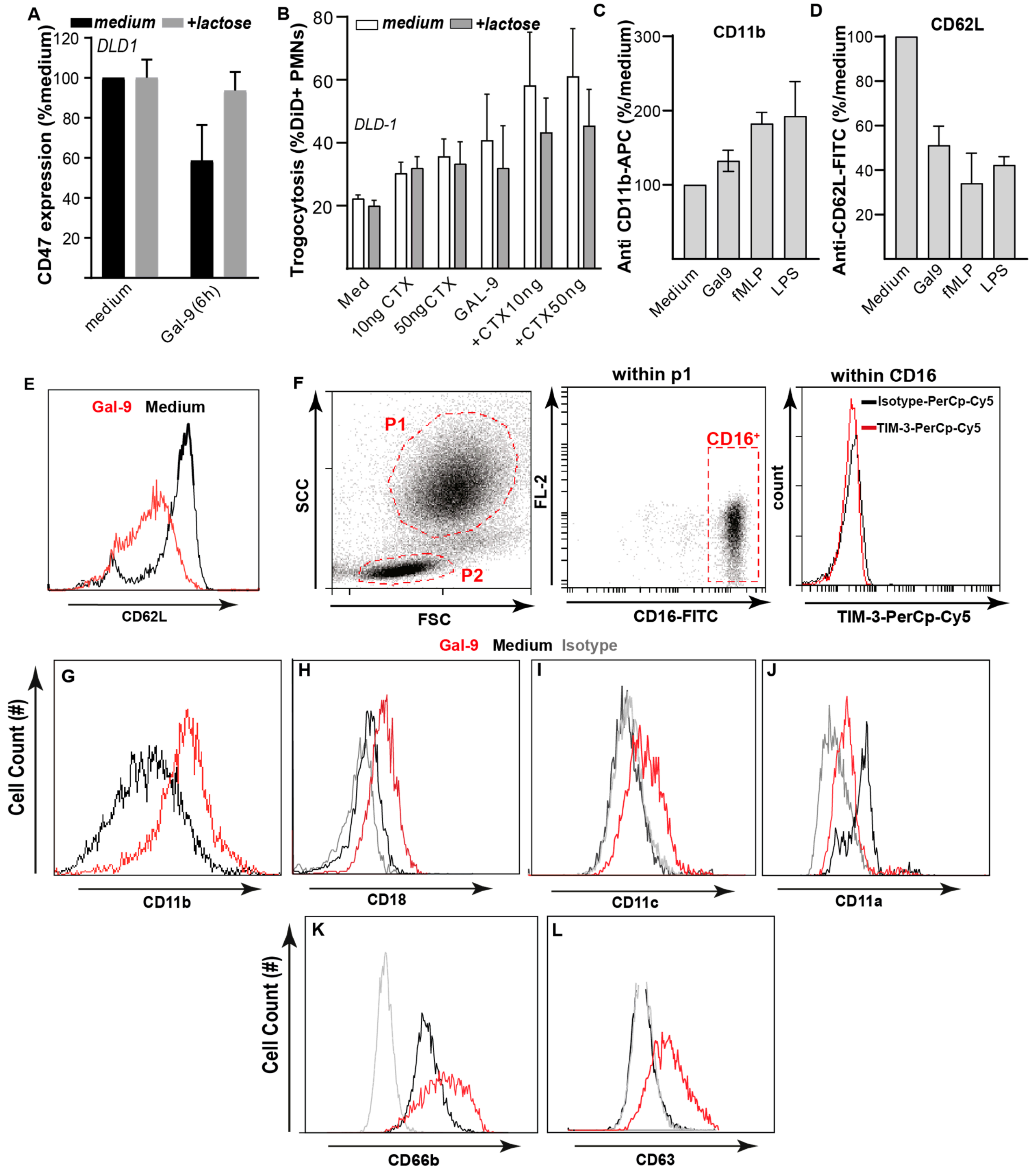

Figure S1. Gal-9-mediated trogocytosis can largely be attributed to neutrophil activation. (**A**) Blockade of Gal-9 mediated expression of PSCD47 on FaDu with α-lactose. (**B**) Gal-9 pre-treated DLD-1 potentiates antibody-dependent cellular phagocytosis (ADCP) with CTX. (**C-E**) Expression of the activation markers CD11b and CD62L on neutrophils after treatment with Gal-9/fMLP/LPS. (**F**) TIM-3 expression on neutrophils. (**G-L**) Expression of different granules markers on Gal-9 treated neutrophils.

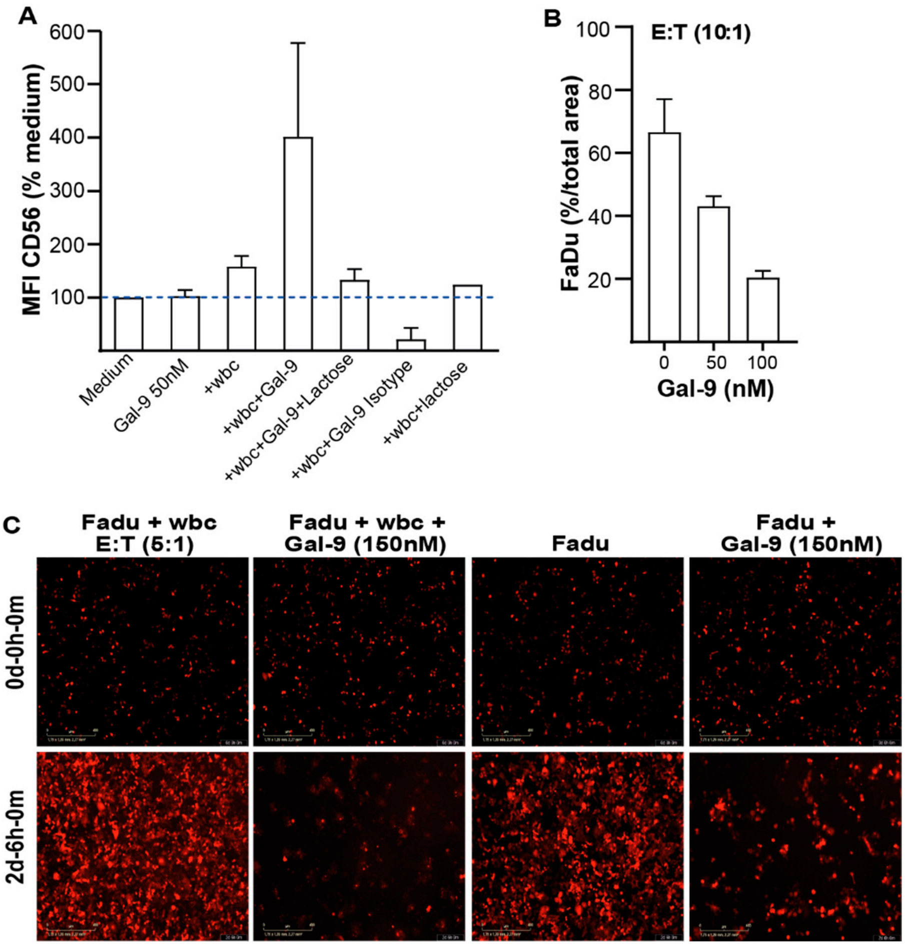

Figure S2: **Gal-9 mediated the cytotoxic elimination of different carcinoma cell lines by leukocytes.** (**A**) ICAM-1 expression on FaDu as determined by flow cytometry in a mixed culture with neutrophils and Gal-9. (**B-C**) Viability of FaDu in a mix culture with Gal-9 and neutrophils. (**C**) Viability of FaDu Flip-GFP Mcherry in a mix culture with Gal-9 and neutrophils.
